# Proteomic approaches for profiling negative fertility markers in inferior boar spermatozoa

**DOI:** 10.1038/srep13821

**Published:** 2015-09-08

**Authors:** Woo-Sung Kwon, Shin-Ae Oh, Ye-Ji Kim, Md Saidur Rahman, Yoo-Jin Park, Myung-Geol Pang

**Affiliations:** 1 Department of Animal Science and Technology, Chung-Ang University, Anseong, Gyeonggi-do 456-756, Korea

## Abstract

The ability to predict male fertility is of paramount importance for animal breeding industries and for human reproduction. Conventional semen analysis generally provides information on the quantitative parameters of spermatozoa, but yields no information concerning its functional competence. Proteomics have identified candidates for male fertility biomarkers, but no studies have clearly identified the relationship between the proteome and sperm fertility. Therefore, we performed a proteomic analysis to investigate small and large litter size boar spermatozoa and identify proteins related to male fertility. In this study, 20 proteins showed differential expression levels in small and large litter size groups. Nineteen of these proteins exhibited decreased expression in large litter size samples and increased expression in the small litter group. Interestingly, only one protein was highly expressed in the large litter size spermatozoa. We then identified signaling pathways associated with the differentially expressed protein markers. Glutathione S-transferase Mu3 and glutathione peroxidase 4 were related to the glutathione metabolic pathway and arginine vasopressin receptor 2 was linked to vasopressin R2/STAT. In summary, this is the first study to consider negative fertility biomarkers, and the identified proteins could potentially be used as biomarkers for the detection of inferior male fertility.

The ability to predict male fertility is of paramount importance for breeding animal herds when artificial insemination is involved^1^. Conventional semen analyses generally provide information on quantitative parameters, including the percentage of motile spermatozoa, the percentage of spermatozoa with normal morphology, and the concentration in a unit dose. While these assays provide valuable quantitative data, they yield no information concerning the functional competence of the spermatozoa^2^,^3^. Moreover, there are many difficulties in the design of studies that assess the value of traditional sperm parameters. These difficulties include determining the number of semen samples to assess and the relevance of different physiological endpoints^4^. Traditional semen analysis is therefore only a limited first-line tool in the diagnosis of male fertility. The value of traditional semen parameters in the diagnosis and prognosis of male fertility has been debated for almost 60 years, and the debate continues^4^,^5^.

Proteomics is a key area of emerging research in the post-genomic era^6^,^7^,^8^. Proteomics can be defined as the qualitative and quantitative comparison of proteomes to identify the cellular mechanisms that are involved in biological processes^6^. As proteins are responsible for cellular function, it is critical to perform comprehensive and systematic identification and quantification of proteins expressed in cells and tissues to gain new insights into these processes^6^. Advances in two-dimensional electrophoresis (2-DE) for the separation of proteins and, in particular, mass spectrometry (MS) for peptide sequencing to facilitate protein identification, has led to the rapid expansion of this field^6^,^9^,^10^,^11^,^12^. Recently, proteomic studies have been performed to identify biomarkers associated with fertility^9^,^10^,^12^. As such, comparative analyses of sperm proteomes are having a major impact on the understanding of how spermatozoa acquire their capacity for fertilization and why spermatozoa have varying levels of fertility^10^,^11^,^12^. However, proteomics-based studies have intrinsic strengths and weakness, and a more sensitive approach is required to accurately explain the relationship of specific proteins with male fertility.

Although several studies have reported the identification of fertility-related biomarkers, a full understanding of these is lacking, and the choice of methods to use to make an accurate prognosis and diagnosis of male fertility is controversial. Proteomic analyses of bovine spermatozoa have identified several proteins that show a correlation with bovine fertility, but field trials to confirm the findings have not been done^10^. Moreover, Kwon and colleagues^12^ reported the discovery of several markers that predict boar fertility. However, the discovered biomarkers were in reality able to predict superior litter size, because their use of the term ‘low-litter size’ was equivalent to an average litter size according to Landrace^13^. Therefore, to discover negative fertility biomarkers for more accurate prognosis and diagnosis of male fertility, we employed a comprehensive and comparative proteomic analysis using boar spermatozoa that had generated small (below average) and large (average) litter sizes^13^. Additionally, to understand the molecular functions of the proteins detected by 2-DE, we identified the signaling and metabolic pathways that the differentially expressed proteins participate in.

## Results

### Proteome profiles of boar spermatozoa

**Figure 1:**
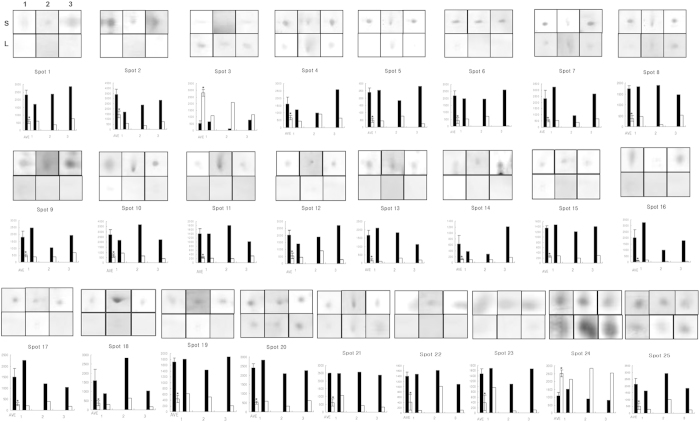
Differentially expressed spots in small and large litter size spermatozoa by 2-D electrophoretic separation. Spots in boxes (upper panels) indicate individual spots from 3 replicates of small and large litter size spermatozoa. The symbols indicate replicate and litter size (S = Small litter size, L = Large litter size, 1 = First replicate gel, 2 = Second replicate gel, 3 = Third replicate gel). Graphs (lower panels) indicate spot densities. Data represent mean ± SEM, n = 3. Protein expression ratios denoted with an asterisk were significantly different (^*^P < 0.05).

To analyze differences in male fertility at the protein level, we compared the protein expression profiles of spermatozoa from boars that yielded small and large litter sizes. 2-DE analysis revealed differences among proteins between low and high fertility groups (Fig. 1, Supplementary Fig. S1). It was important to assess the level of variation in the 2-DE evaluations of boar spermatozoa. To address this issue, detailed gel analyses were performed using the SigmaGel software (Jandel Scientific, San Rafael, CA) on three paired experiments (Fig. 1, Supplementary Fig. S1). Figure 1 depicts the 2-DE gels of spermatozoa from small and large litter size samples and illustrates that the majority of the protein spots were significantly different between the groups (>4-fold). We identified 357 ± 21 and 328 ± 17 protein spots in spermatozoa from small and large litter size groups, respectively. Because the standard errors of protein spot numbers for each group were not high, we suggest that possible variations have been minimized. Approximately 102 protein spots were consistently observed in each sample. Of these 102 spots, 25 spots showed differential expression in intensity between small and large litter size groups. There were 2 spots with decreased expression in the small litter size sample and increased expression in the large size group; 23 spots were decreased in the large litter size sample but increased in the small size group. One of the two proteins highly expressed in large litter size sperm, and 19 of the 23 proteins highly expressed in small litter size sperm, were identified by LC-MS/MS analysis (spots no. 1, 11, 15, 19, and 24 were not identified, Table 1).

### Immunolocalisation of glutathione peroxidase 4 (GPx4) and arginine vasopressin receptor 2 (AVPR2) in spermatozoa

**Figure 2:**
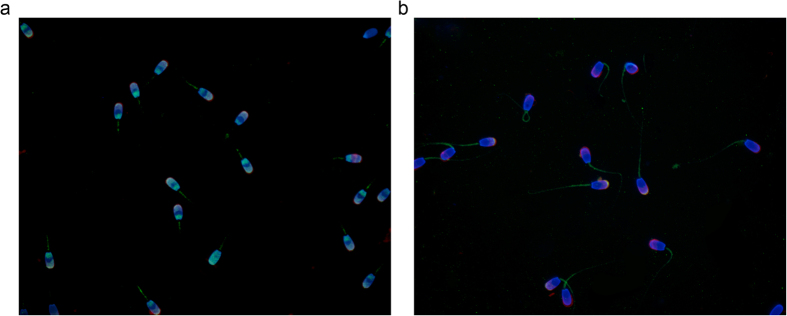
Localization of GPx4 and AVPR2 in boar spermatozoa. (**A**) Merged image of nucleus (DAPI, blue) and the acrosome (lectin PNA, red), and GPx4 (green). (**B**) Merged image of the nucleus, the acrosome, and AVPR2 (green). Images were obtained using a Nikon TS-1000 microscope and NIS Elements software (Nikon, Japan).

To examine the localization of GPx4 and AVPR2 proteins in boar spermatozoa, immunofluorescence was conducted using antibodies for GPx4 and AVPR2, lectin PNA, and DAPI. GPx4 was localized to both the head and midpiece of the spermatozoa (Fig. 2A). The AVPR2 protein was expressed in the tip of the acrosomal region and in the midpiece (Fig. 2B). Lectin PNA co-stained in the acrosome with GPx4 and AVPR2, respectively.

### Protein confirmation by western blotting

**Figure 3:**
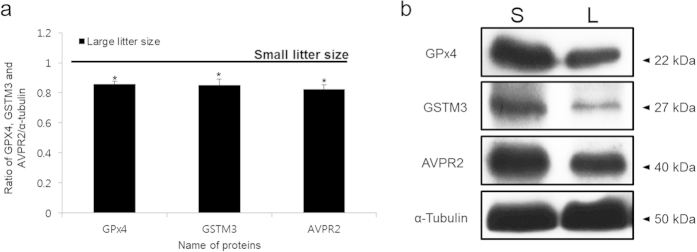
Expression of GSTM3, GPx4 and AVPR2 in small and large litter size spermatozoa. (**A**) Ratios of GPx4, GSTM3 and AVPR2 [optical density (OD × mm)/α-tubulin (OD × mm)] in small and large litter size spermatozoa. The black line indicates small litter size. Data represent mean ± SEM, n = 3. Protein expression ratios denoted with an asterisk were significantly different (^*^P < 0.05). (B) GPx4, GSTM3, and AVPR2 were probed with anti-GSTM3, anti-GPx4, and anti-AVPR2 antibodies. (S = Small litter size, L = Large litter size).

Western blotting was performed to validate the 2-DE results. GPx4, glutathione S-transferase Mu3 (GSTM3), and AVPR2 were detected at positions corresponding with ~22, 27 and 40 kDa, respectively. The intensity of the GPx4, GSTM3, and AVPR2 bands was significantly greater in the small litter size samples (*P* < 0.05, Fig. 3).

### Signaling pathway analysis

**Figure 4:**
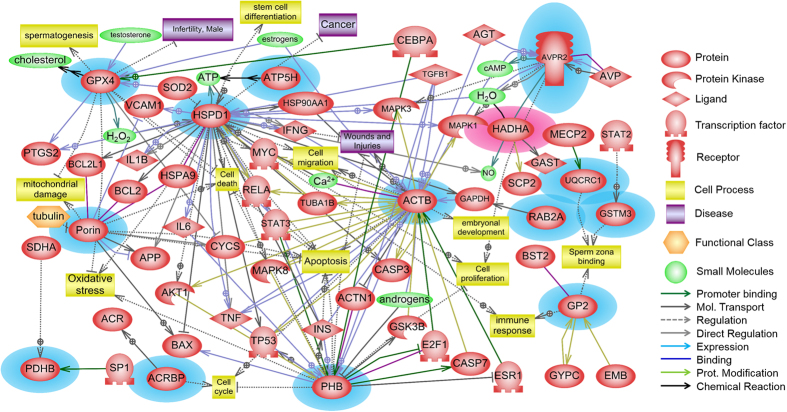
Signaling pathways associated with fertility-related proteins in boar spermatozoa. The schematic was established using Pathway Studio 9.0 following a database search in PubMed. The red-highlighted protein was highly expressed in large litter size spermatozoa, while the blue-highlighted proteins were highly expressed in small litter size spermatozoa.

Signaling pathways were identified using Pathway Studio to understand the signaling and metabolic pathways associated with differentially expressed protein markers. Two significant networks, metabolic and receptor-signaling pathways, were identified (*P* < 0.05, Table 2). Glutathione metabolism was the significant pathway associated with GPx4 and GSTM3 (*P* < 0.05, Table 2). In addition, the vasopressin R2/STAT signaling pathway was associated with AVPR2. A schematic image was created to determine both physiological function and interactions of the proteins that were expressed differentially in the small and large litter size spermatozoa. The differentially expressed proteins were found to interact with each other or other proteins, and were also related to particular protein kinases, ligands, transcription factors, receptors, cell processes, diseases, functional classes, and small molecules (Fig. 4). At least 14 proteins were implicated among the 20 identified proteins (Fig. 4).

## Discussion

As proteins, or more correctly protein-protein interactions, are responsible for cellular function, it is critical that a comprehensive and systematic identification and quantification of proteins expressed in cells and tissues is undertaken to gain insight into these processes. The aim of the present study was to identify proteins that were differentially expressed between small and large litter size boar spermatozoa for accurate determination and prediction of levels of male fertility. Proteomic approaches were used to identify fertility-related proteins in the spermatozoa of low fertility boars, and signaling pathways associated with fertility-related proteins were identified.

Generally, protein spots were selected based on intensity differences. These differences in previous studies were 3-fold at most^10^,^11^,^12^. However, increased or decreased expression was defined as a greater than 4-fold difference in the present study. Our results were consistent with a previous human study, in which infertile patients showed increased levels of protein expression compared to normal fertile controls^14^. In the present study, we found several proteins related to phosphorylation, including mitochondrial trifunctional proteins (HADHA); stress-related proteins, including 60 kDa heat shock protein; acrosome reaction-related proteins, including acrosin-binding protein precursor and GSTM3; antioxidant related proteins, including GPx4; glycolysis/citric acid cycle-related proteins, including pyruvate dehydrogenase (PDHB); and AVPR2. A previous report of a comparative proteomic analysis of high fertility and low fertility in bulls identified protein interaction networks for 125 putative biomarkers that included predicted and hypothetical proteins^10^,^15^.

In the present study, 20 differentially expressed proteins were identified between small and large litter sizes in pigs. HADHA, composed of 4 alpha and 4 beta subunits, catalyzes 3 steps in the mitochondrial beta-oxidation of fatty acids that represent activities of long-chain 3-hydroxyacyl-CoA dehydrogenase, long-chain enoyl-CoA hydratase, and long-chain thiolase^16^. HADHA subunits are encoded by different nuclear genes located on chromosome 2 in human^16^. In HADHA protein deficiency, all three mitochondrial trifunctional protein enzymes are deficient, resulting in impaired energy production from fat and accumulation of long-chain hydroxy-acyl-CoA and long-chain hydroxy-acylcarnitine upstream of the enzyme block^16^,^17^. Clinically, classic trifunctional protein deficiency usually results in sudden unexplained infant death, a Reye-like syndrome, cardiomyopathy, and/or skeletal myopathy^18^. This was the only one of 20 identified proteins that showed increased expression in the large litter size group. This protein was detected on 2-DE gels, but signaling pathway analysis using Pathway Studio showed no significant association with sperm fertility. As such, the effect of this protein on spermatozoa and male fertility remains unclear. However, the trifunctional protein mediates thyroid hormone receptor-dependent stimulation of mitochondria metabolism^19^, and mitochondria perform a very important role in sperm motility. Mitochondrial proteins downregulated under glucotoxic conditions include voltage-dependent anion-selective channel protein 2 (VDAC2)^20^. VDAC2 was previously reported as a highly expressed protein in low-fertility bulls^10^. Moreover, Kwon *et al.*^21^ reported that VDAC2 levels do affect male fertility. From these results, one could infer that HADHA in large litter size sperm downregulates VDAC2 to affect male infertility.

GPx4 is known as an antioxidant enzyme that is involved in sperm motility and is abundantly expressed in sperm of normal fertility before capacitation^22^. Interestingly, GPx4 was more highly expressed in small litter size sperm in this study. GPx4 prevents apoptotic cell death through suppression of reactive oxygen species (ROS) generation^23^,^24^. Although the excessive ROS production in spermatozoa induces abnormal function and cell death, moderate amounts of ROS are indispensable for capacitation^25^. These facts suggest that the excessive generation of GPx4 protein of spermatozoa leads to infertility through enhanced suppression of ROS production. In addition, the results of signaling pathway analysis showed that GPx4 protein has an association with sperm fertility, specifically through the glutathione metabolism pathway (Table 2).

The glutathione S-transferases (GSTs) are a group of polymorphic enzymes that are important for protecting against oxidative stress. A sperm-specific role for GST would be the detoxification function contributed by GSTM proteins, which, by eliminating reactive oxygen species via glutathione, prevents lipid membrane peroxidation, a process highly damaging to sperm membrane integrity^26^. However, GSTM was also identified as a small ubiquitin-like modifiers (SUMO) target in sperm. SUMO is another type of post-translational modification in sperm. Non-motile, two-tailed, curled-tailed, misshapen, microcephalic (small head) and acephalic (no head) sperm exhibited abnormally high levels of SUMOylation in their neck and tail regions relative to normal sperm^27^. Thus, SUMOylation is involved in stress responses *in vivo* and *in vitro* in mouse testicular cells^28^, and stress-related proteins, including GSTM proteins, were additionally identified as SUMO targets^27^. SUMOylation of SUMO targets may explain why GSTM3 in this study was highly expressed in the small litter size group spermatozoa, despite a role in protection of sperm from oxidative stress for GSTM3.

AVPR2 was localized to the acrosome region and midpiece in cauda epididymal spermatozoa, but was only found in the midpiece in caput epididymal spermatozoa^29^. Neurohypophysial hormones, such as arginine vasopressin (VP), affect the male reproductive tract. VP stimulates male duct smooth muscle contraction and potentiates the smooth muscle contraction that is induced by adrenergic agonists^30^,^31^,^32^. In other words, VP has been implicated in stimulating contractile activity of the male reproductive tract in the testis. Thus, neurohypophysial hormones appear to play an important, although not fully understood, role in male fertility. In particular, AVPR2 mRNA was found in the vas deferens epithelium^33^, and higher levels of VP decrease sperm count and motility in mouse^29^. It is already known that VP affects sperm fertility and sperms have VP receptors, suggesting that the number of receptors is related with sperm fertility. Indeed, AVPR2 levels were increased in the small litter size group, according to the 2-DE analysis in this study. Furthermore, this study confirmed that AVPR2 expressed their function via the vasopressin R2/STAT signaling, one of the receptor signaling pathways found to be associated with sperm fertility using Pathway Studio software. This result is consistent with the report of Hagedorn *et al.*^33^, which showed that VP has the potential to acutely change the environment to which sperm are exposed and, thus, has the potential to affect male fertility.

It was recently reported that Ras-related protein Rab-2, GPx4, PDHB, and ACRBP were associated with capacitation^11^. Moreover, Kwon *et al.*^12^ showed that Ras-related protein Rab-2A (RAB2A) was highly expressed in average litter size spermatozoa. RAB2A is involved in acrosome formation and regulates vesicular transport and membrane fusion^34^,^35^. Acrosin-binding protein (ACRBP) plays a role in the capacitation pathway and regulates acrosin release by the acrosome^36^,^37^. Pyruvate dehydrogenase (PDHB), one of the subunits of the pyruvate dehydrogenase complex, plays a role in energy metabolism and tyrosine phosphorylation during capacitation^38^,^39^,^40^. Moreover, other proteins (mutant beta-actin, actin-related protein T2, actin-related protein T3, glutathione S-transferase Mu3, porin, prohibitin, cytochrome b-cl complex subunit 1, isoform2, mitochondrial, ATP synthase subunit d, mitochondrial, and 60 kDa heat shock protein, mitochondrial) are involved in determining the structure of spermatozoa, ROS metabolism, energy metabolism, or the stress response^11^,^41^,^42^,^43^,^44^,^45^,^46^,^47^. Additionally, new signaling pathways were established to understand the interactions of these proteins with others at the cellular level (Fig. 4). The signaling pathway identified indicated that these differentially expressed proteins hypothetically control male fertility through interactions with a variety of factors.

To our knowledge, differentially expressed proteins that play critical roles in spermatozoa might be key factors for male fertility. Therefore, we suggest that these biomarkers may be useful for prognosis and diagnosis of male fertility. Moreover, we anticipate that proteins identified in the present study may be used as negative biomarkers for detection of inferior male fertility, such as sub-fertility or infertility. While we do not yet know the complete roles of the differentially expressed proteins, this study represents a useful basis for further investigation. Specifically, these results will enable further elucidation of the molecular mechanisms involved in this particular condition and might shed further light on key sperm proteins involved in fertilization. This study is also an important prerequisite to the development of diagnostic tests to identify low fertility in men in a clinical environment, and, in the future, might also be applied to the development of novel methods of sperm-targeted contraception.

## Methods

### Semen sources and preparation

All procedures were performed according to guidelines for the ethical treatment of animals and were approved by the Institutional Animal Care and Use Committee of Chung-Ang University. Boar spermatozoa have been used as an ideal model for male fertility, because they provide several advantages such as clear records of pregnancy success or failure and litter size^48^. Therefore, we used Duroc boar semen samples as a model for male fertility in mammals. Boar fertility data were obtained from Darby Genetics, Inc. (Gyeonggi-do, Korea). Litter size was used as the *in vivo* fertility parameter. To evaluate fertility, we considered more than 5 insemination events and more than 5 litters from each boar (on average, 22.46 inseminations and 8.96 litters per boar), and 24 boars were ultimately ranked by litter size^49^. The average litter size for each boar was calculated from the total number of piglets born from each farrowing, averaged across all farrowings^50^. To avoid individual male factors, the 9 lowest-ranked (on average 6.23 ± 0.38 litters) and the 9 highest-ranked (on average 10.99 ± 0.01 litters) were randomly divided and pooled into three groups in small and large litter sizes for experimental replication (n = 3)^11^,^12^. Samples were prepared for proteomic study as previously described^11^,^12^.

### Two-dimensional electrophoresis (2-DE)

Sperm samples were solubilised in rehydration buffer (7 M urea, 2 M thiourea, 4% CHAPS (w/v), 0.05% Triton X-100, 24 μM PMSF, 1% octyl β-D-glucopyranoside, 20 mM DTT, 0.5% IPG buffer and 0.005% bromophenol blue) for 1 h at room temperature. Insoluble materials were removed by centrifugation at 20,000 × g for 10 min. The supernatant (350 μl equivalent to 5 × 10^8^ cells containing 150 μl proteins) was added to an IPG strip holder, overlaid with an IPG Strip (18 cm, pH 3–10 linear), and subjected to isoelectric focusing with and IPGphor (Amersham Bioscience; rehydration; 12 h, 200 V; 1 h, 500 V; 1 h, 1000 V; 1 h, from 8000 V to 8800 V). The focused strip was rotated in 10 ml 65 mM DTT in equilibration buffer (50 mM Tris-HCl, pH 8.8, 6 M urea, 30% (v/v) glycerol, 2% (w/v) SDS, 0.005% (w/v) bromophenol blue) for 15 min, and subsequently in 135 mM iodoacetamide in 10 ml equilibration buffer for 15 min. Proteins were separated by SDS-PAGE using pre-cast gradient gels and a horizontal Multiphore gel system (Amersham Bioscience; 130 V, 180 mA for 12 h). Molecular weight markers were also run on each gel (0.3 μl), and pI values were assigned from known proteins using broad range unstained protein markers (LandMark^TM^) as internal standards on a reference gel.

### Gel analysis

Proteins were visualized by silver staining with a standard protocol^11^,^12^, except that glutaraldehyde was omitted from the sensitizing step and formaldehyde was excluded from the silver solution but included in the developing step. Gels were initially fixed in 40% (v/v) ethanol, 10 (v/v) acetic acid, for 30 min, and then sensitized in 0.2% (w/v) sodium thiosulphate, 30% (v/v) ethanol, 0.83 M sodium acetate, for 30 min. Following three washes in distilled water, the gel was subjected to the silver reaction (0.25% (w/v) silver nitrate) for 20 min and subsequently washed twice more. The gels were developed in 0.24 M sodium carbonate, 0.015% (w/v) formaldehyde for 1 min and then for 3 min with a fresh solution. Development was quenched with 50 mM EDTA for 10 min and the gel was stored in 1% (v/v) acetic acid^51^,^52^. Gels were scanned at a high resolution (500 dpi) with a high specification scanner and saved as TIFF images. Detailed gel analysis was subsequently performed using the Sigma Gel program (Sigma, USA). The software automates the identification and quantification of gel spots by normalizing spot volumes and excluding background noise spot according to a minimum area parameter. Pairs of 2-DE gels can be compared with the software, which matches spots between gels by comparing their location (selected by determining the centre of optical density within each spot). It was also necessary to manually confirm that each spot had been identified correctly by the software and to validate the results for spot comparison between gels. In this study, the marked difference in spot intensity was set as a ≥ 4-fold difference following advice from the software manufacturers^14^.

### Protein digestion

The dried protein fractions were dissolved and reduced in 10 μl solution containing 8 M urea, 5 mM EDTA, 10 mM TCEP, and then incubated at room temperature for about 2 h. To avoid complications with over-alkylation, 15 cysteines were not ‘capped’, and reformation of disulfides was prevented by the continued presence of stable TCEP. The reduced proteins were diluted to 40 μl with 50 mM ammonium bicarbonate, pH 7.8, to give a final urea concentration of 2 M. Sequencing grade trypsin (20 pmol) (V5111, Promega, Madison, WI) was added to the protein solution and the digestion was performed overnight at 37 °C.

### LC-MS/MS Analysis

The tryptic peptides digested from proteins were analyzed using a liquid chromatography system coupled to the nanoflow electrospray source of a QTOF II (Micromass, UK) mass spectrometer. Peptides were separated on a C18 reverse phase column (0.15 mm × 150 mm, VC-10-C18–150; Micro-Tech Scientific, Vista, CA, USA) using a binary solvent system made up of 98.8% water, 1% acetonitrile, and 0.2% formic acid (solvent A), and 94.8% acetonitrile, 5% water, and 0.2% formic acid (solvent B). The peptides were eluted from the column with a linear gradient program that changed the composition of solvent from 5% B to 90% B over 100 min at a constant flow rate of 1 μl/min. Data from the eluted peptides were acquired using the Waters MassLynx 4.0 software. An initial 40 min MS/MS analysis (1 μl injected from each fraction) was performed to check the quality and the concentration of the secretome. Final data were acquired using 180 min analyses (approximately 2 μg protein injected based on the intensity of the initial run) in a data-dependent mode, where each full MS scan was followed by MS/MS of the four most intense ions. To optimize peptide coverage, a mass/charge exclusion list was maintained so that the same peptide was not selected for MS/MS within a period of 1 min. To confirm the reliability of the MS results, the analysis of two preparations from each sample was duplicated.

### Database searching

These data were then used to search the NCBI non-identical protein sequence database using MASCOT software (Matrix Science), and statistically significant hits were recorded together with the number of peptides and percentage coverage of the protein.

### Immunolocalisation of GPx4 and AVPR2

To determine protein localization in spermatozoa, we used an immunocytochemistry approach according to our previous study^11^,^21^,^29^. Briefly, the spermatozoa were washed twice in Dulbecco’s phosphate-buffered saline (DPBS) and the pellets were resuspended with 1 ml DPBS. The suspensions were smeared onto glass slides and allowed to air-dry. Then, the slides were fixed in 3.7% paraformaldehyde (PFA) for 30 min at 4 °C. After fixation, the slides were washed with PBS and then washed twice in PBS containing 0.1% Tween-20 (PBS-T). To block non-specific binding sites, the slides were first incubated with PBS-T containing 5% BSA for 1 h. Slides were then incubated overnight with polyclonal anti-GPX4 rabbit antibody (Abcam, Cambridge, UK) or polyclonal anti-AVPR2 rabbit antibody (Acris, Herford, Germany) diluted 1:100 in PBS-T containing 5% BSA for 1 h. After two washes with PBS-T and PBS, the slides were incubated at room temperature in the dark for 2 h with an anti-rabbit FITC-conjugated IgG (Abcam, Cambridge, UK) diluted 1:3000, and lectin PNA-conjugated Alexa Fluor 647 (Molecular Probes, Eugene, OR, USA) diluted 1:100 to stain the acrosome region. Following 2-h incubation, the slides were washed with PBS and counter-stained with 1 mg/ml DAPI diluted 1:3000 in PBS and sealed with a cover slip.

### Western blotting

To validate 2-DE results, expression levels of GPx4, GSTM3, and AVPR2 were analyzed by western blotting as described^11^,^12^,^41^. Samples were washed with DPBS by centrifugation at 10,000 × *g* for 10 min after incubation. Sperm pellets were resuspended in Laemmli sample buffer (63 mM 1 Tris, 10% glycerol, 10% sodium dodecyl sulfate, 5% bromophenol blue) containing 5% 2-mercaptoethanol and incubated for 10 min at RT. Finally, the supernatants were separated by centrifugation at 10,000 × *g* for 10 min and boiled for 3 min at 100 °C. Samples were subjected to SDS-polyacrylamide gel electrophoresis using a 12% mini-gel system (Amersham, Piscataway, NJ, USA), and the separated proteins were transferred to a membrane. GPx4, GSTM3, and AVPR2 were detected by incubation with polyclonal anti-GSTM3 rabbit antibody (LSBio, Seattle, WA), polyclonal anti-GPX4 rabbit antibody (Abcam, Cambridge, UK) and polyclonal anti-AVPR2 rabbit antibody (Acris, Herford, Germany), each diluted to 1 μg/ml with blocking solution, for 2 h at RT, respectively. Next, α-tubulin was detected by incubation with monoclonal anti-α-tubulin mouse antibody (Abcam) diluted 1:2000 with 5% blocking agent for 2 h at RT. The membranes were then incubated with horseradish peroxidase-conjugated anti-rabbit IgG and anti-mouse IgG (Abcam) for 1 h at RT. Proteins on the membrane were visualized using an enhanced chemiluminescence (ECL) technique. All bands were scanned with a GS-800 Calibrated Imaging Densitometer (Bio-Rad, Fremont, CA, USA) and analyzed using Quantity One software (Bio-Rad, Hercules, CA, USA). The density of the bands was quantified according to the α-tubulin ratio (GPx4, GSTM3, and AVPR2/α-tubulin).

### Signaling pathway analysis

Pathway Studio (v9.0, Ariadne Genomics, Rockville, MD) was used to investigate the relevant molecular function of novel fertility-related protein markers in small and large litter size boar spermatozoa. Protein lists were imported into Pathway Studio to identify the cell processes influenced by these proteins. Identified metabolic or signaling pathways were confirmed via the PubMed/Medline hyperlink embedded in each node. New signaling pathways were created to visualize interactions between differentially expressed proteins.

### Statistical analysis

The experimental data were expressed as the mean ± the standard error of the mean (SEM). Data were analyzed with an independent sample *t*-test or one-way analysis of variance (ANOVA) followed by Duncan’s Multiple Range test using SPSS 18.0 for Windows (Chicago, IL, USA). The probabilities of the signaling pathways were determined using the Fisher exact test. A *P* value < 0.05 was considered to be statistically significant.

## Additional Information

**How to cite this article**: Kwon, W.-S. *et al.* Proteomic approaches for profiling negative fertility markers in inferior boar spermatozoa. *Sci. Rep.* **5**, 13821; doi: 10.1038/srep13821 (2015).

## Supplementary Material

Supplementary Information

## Figures and Tables

**Table 1:** Differentially expressed proteins in small and large litter size spermatozoa.

Spot No.	NCBI No.	Protein description	MASCOT score^a^	Expression level	
				Small litter size	Large litter size
2	1127023	pancreatic glycoprotein 2 (GP2)	158	▴	▾
3	47522754	Trifunctional enzyme subunit alpha, mitochondrial (HADHA)	202	▾	▴
4	359811347	60 kDa heat shock protein, mitochondrial (HSPD1)	243	▴	▾
5	73985642	Cytochrome b-cl complex subunit 1, isoform2, mitochondrial (UQCRC1)	148	▴	▾
6	28336	mutant beta-actin (beta’-actin) (ACTB)	107	▴	▾
7	57086887	actin-related protein T2 (ACTRT2)	205	▴	▾
8	84579847	actin related protein T3 (ACTRT3)	197	▴	▾
9	3891849	Chain B, crystal structure of bovine mitochondria	105	▴	▾
10	300763221	pyruvate dehydrogenase: SUBUNIT = beta precursor (PDHB)	155	▴	▾
12	4929676	Homo sapience CGI-104 protein mRNA, complete cds	236	▴	▾
13	109102081	cytosolic 5′ nucleotidase IB isoform 1	87	▴	▾
14	75052483	Acrosin-binding protein precursor (Proacrosin-binding protein sp32) (ACRBP)	335	▴	▾
16	190201	Porin	48	▴	▾
17	4505773	Prohibitin (PHB)	207	▴	▾
18	114053087	glutathione S-transferase Mu3 (GSTM3)	336	▴	▾
20	27807305	ATP synthase subunit d, mitochondrial (ATP5H)	145	▴	▾
21	311253799	Ras-related protein Rab-2A (RAB2A)	59	▴	▾
22	76779289	glutathione peroxidase 4 (GPx4)	147	▴	▾
23	13195731	glutathione peroxidase 4 (GPx4)	241	▴	▾
25	28417	Arginine vasopressin receptor 2 (AVPR2)	203	▴	▾

^a^MASCOT score is −10Log(p), where p is the probability that the observed match is a random event. Scores >55 indicate identity or extensive homology (*P* < 0.05).

**Table 2:** Signaling pathways associated with differentially expressed protein markers.

Signaling pathway	Function	Overlapping Entities	p-value
Metabolic Pathway	Glutathione metabolism	GSTM3, GPx4	0.01079
Receptor Signaling Pathway	Vasopressin R2/STAT signaling	AVPR2	0.01687
