# Supplementary material for: Proteomic approaches for profiling negative fertility markers in inferior boar spermatozoa
Source: Sci Rep. 2015 Sep 8;5:13821. doi: 10.1038/srep13821 (PMC4562270; doi:10.1038/srep13821)

Supplementary data

Proteomic approaches for profiling negative fertility markers in inferior boar spermatozoa

Woo-Sung Kwon, Shin-Ae Oh, Ye-Ji Kim, Md Saidur Rahman, Young-Ah You, and Myung-Geol Pang

Supplementary information includes:

Supplementary Figure S1

Supplementary Figure S1. Two-D electrophoretic separation of sperm proteins from the small and large litter size spermatozoa. S-1, S-2 and S-3 are small litter size, and L-1, L-2 and L-3 are large litter size. Proteins from 5 × 10^6 cells were loaded onto each gel. The apparent molecular weights are indicated down the side, and the pH range across the lower end of the gels. All experiments carried out three replications. Spots showing include increased/decreased or missing protein expression in small and large litter size gels. 25 difference spots are indicated arrows.

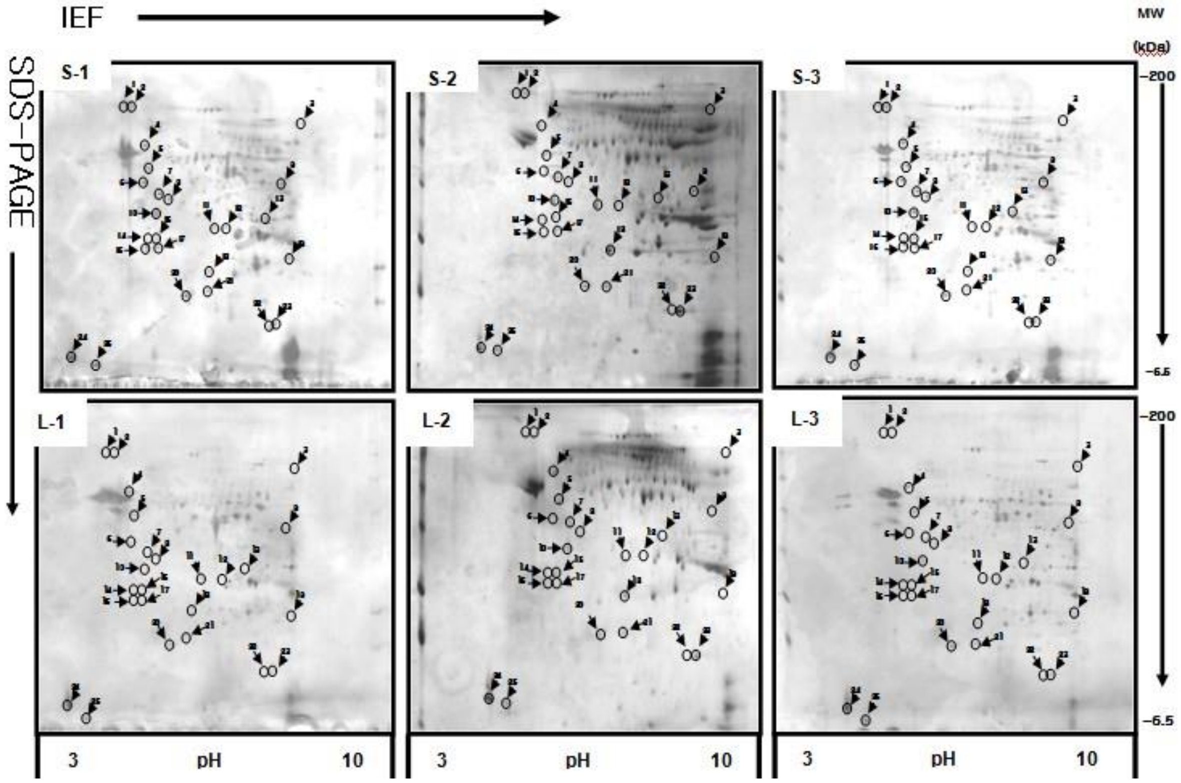

Supplement: Supplementary Information [file srep13821-s1.pdf]
